# Cumulative Epigenetic Abnormalities in Host Genes with Viral and Microbial Infection during Initiation and Progression of Malignant Lymphoma/Leukemia

**DOI:** 10.3390/cancers3010568

**Published:** 2011-02-04

**Authors:** Takashi Oka, Hiaki Sato, Mamoru Ouchida, Atae Utsunomiya, Tadashi Yoshino

**Affiliations:** 1 Department of Pathology, Graduate School of Medicine, Dentistry and Pharmaceutical Sciences, Okayama University, 2-5-1 Shikata-cho, Okayama 700-8558, Japan; E-Mail: yoshino@md.okayama-u.ac.jp; 2 Department of Medical Technology, Graduate School of Health Science, Okayama University Medical School, 2-5-1 Shikata-cho, Okayama 700-8558, Japan; E-Mail: hiaki@md.okayama-u.ac.jp; 3 Department of Molecular Genetics, Graduate School of Medicine, Dentistry and Pharmaceutical Sciences, Okayama University, 2-5-1 Shikata-cho, Okayama 700-8558, Japan; E-Mail: ouchidam@md.okayama-u.ac.jp; 4 Department of Hematology, Imamura Bun-in Hospital, 11-23 Kamoike Shinnmachi, Kagoshima, 890-0064, Japan; E-Mail: autsunomiya@jiaikai.jp

**Keywords:** epigenetics, methylation, lymphoma, leukemia, progression, *HTLV-I*, *H Pylori*

## Abstract

Although cancers have been thought to be predominantly driven by acquired genetic changes, it is becoming clear that microenvironment-mediated epigenetic alterations play important roles. Aberrant promoter hypermethylation is a prevalent phenomenon in human cancers as well as malignant lymphoma/leukemia. Tumor suppressor genes become frequent targets of aberrant hypermethylation in the course of gene-silencing due to the increased and deregulated DNA methyltransferases (DNMTs). The purpose of this article is to review the current status of knowledge about the contribution of cumulative epigenetic abnormalities of the host genes after microbial and virus infection to the crisis and progression of malignant lymphoma/leukemia. In addition, the relevance of this knowledge to malignant lymphoma/leukemia assessment, prevention and early detection will be discussed.

## 1. Introduction

Modified DNA bases in prokaryotes are not only used as identification marks to discriminate between self and invading foreign DNA, but also contribute to other functions such as postreplicative mismatch repair [1,2]. Restriction endonuclease and DNA methyltransferase activities are well characterized to recognize certain DNA sequences, enabling bacterial cells to resist infection of bacteriophage or exogenous DNA molecules that are unmethylated or methylated at different recognition sites compared to the host DNA. Such a sequence-specific DNA methylation gives rise to not only inhibition of the restriction endonuclease activity, preserving thereby the integrity of the bacterial genome, but also modulation of the expression of certain bacterial gene sets as well, influencing bacterial virulence [3,4].

In the case of eukaryotes especially in vertebrates, 5-methylcytosine is the predominant modified base in DNA. In mammals, cytosine methylation is essentially confined to the sequence 5′-CpG-3′ [5]. In certain areas of the genome of mammals, a high concentration of these CpG dinucleotides is found, and these are referred to as “CpG islands” (CGIs). DNA methyltransferase I (DNMT1) is known as a “maintenance methyltransferase”, and has been shown to have a 10-fold preference for hemimethylated DNA as a substrate during DNA replication from methylated parental DNA. Mammalian cells mostly use DNMT1 to maintain the DNA methylation profile in a stable fashion through cell division. DNMT3a and DNMT3b are known as “*de novo* methyltransferases” which are used to methylate previously unmethylated DNA during development and differentiation. These DNMTs function to discontinuously change the methylation profile for specific compartments of the genome in a tissue specific manner in vertebrates, in contrast to prokaryotic methyltransferase modifying essentially all of their recognition sites. There are alternating methylated and unmethyated regions in vertebrates and the methylation profiles of the specific compartments may change according to development and tissue-specific differentiation. Methylated CpG islands in the 5′ transcription regulatory region recruits methyl-CpG binding proteins such as MeCP2, MBD1, MBD2 and MBD4, followed by association with histone deacetylase (HDAC) and histone methyltransferase, resulting in formation of a repressive chromatin structure and gene silencing. This may provide “epigenetic memory” by helping progeny cells to “remember” their cellular identity [6]. CpG island methylation is also involved in the regulation of imprinted gene expression and X-chromosome inactivation in addition to the fine-tuning of specific differentiation of cells and the development from stem cells [7-10]. Polycomb group (PcG) and Trithorax group (Trx) proteins are alternative systems of epigenetic memory to regulate gene expression and chromatin structure via modification of histone tails in a heritable manner [11-13]. Transcriptionally active genes in normal cells are marked by hypomethylated promoter CpG islands, histone hyperacetylation and specific histone modifications such as histone H3 lysine 4 (H3K4) di- and tri-methylation and H3K79 methylation. Transcriptionally repressed genes are marked by hypermethylated promoter CpG islands, histone hypoacetylation and H3K9 and H3K27 methylation. The epigenetic landscape of the whole genome is quite different in cancerous cells from that in normal cells. Epigenetic processes have been implicated in the development of various malignancies including lymphoma/leukemia, in which repression or gene silencing of tumor suppressor genes is remarkably common [14-18].

## 2. Epigenetic Alterations Induced by Bacterial Infection

It is estimated that over 15% of malignancies worldwide or about 1.2 million cases per year can be attributed to infections. Infections involving viruses, bacteria and schistosomes have been linked to higher risks of malignancy [19]. In addition to viral infections, bacterial associations to cancer development have been well established. Convincing evidence has linked *Helicobacter pylori* with both gastric cancer and mucosa associated lymphoid tissue (MALT) lymphoma [20,21]. Other species of genus *Bacterium* also associated with cancers include: *Streptococcus bovis* and colon cancer [22,23], *Salmonella typhi* and gallbladder cancer [24,25], and *Chlamydia pneumoniae* with lung cancer [26,27]. Important mechanisms by which bacterial agents were suggested to induce carcinogenesis include chronic infection, immune evasion and immune suppression [22,24,28].

Maternal oral infection, caused by bacteria such as *Campylobacter rectus* or *Porphyromonas gingivalis*, has been implicated as a potential source of placental and fetal infection and inflammatory challenge, which increases the relative risk for pre-term birth and intra-uterine growth restriction. It was revealed that *C. rectus* infection of mice induced hypermethylation in the promoter region-P0 of the imprinting *Igf2* (insulin-like growth factor 2) gene, followed by down regulation of the *Igf2* gene expression. Epigenetic modification of imprinted genes via changes in DNA methylation by bacteria infection was suggested to have a critical effect in fetal growth and development programming [29].

**Figure 1.**
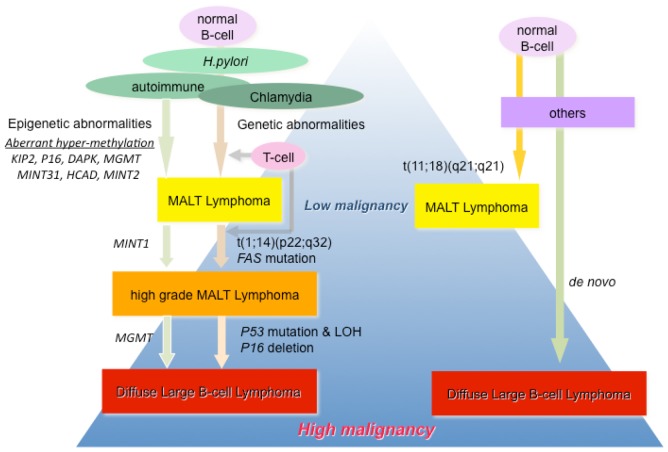
Genetic and epigenetic molecular pathological mechanism of onset and progression of mucosa associated lymphoid tissue (MALT) lymphoma. Arrows indicate genetic or epigenetic events during onset and/or progression of MALT lymphoma.

**Figure 2.**
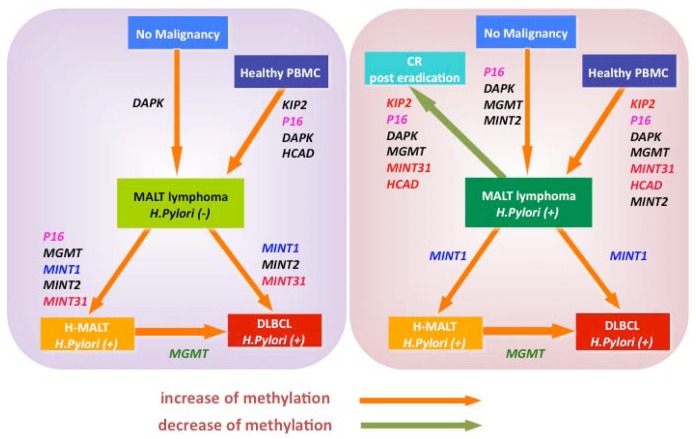
Schematic illustration of development and progression of gastric lymphoma in terms of specific gene methylation. Genes associated with orange arrows indicate significant increase of methylation frequency from arrow start point status to end point status. On the other hand, genes associated with green arrow show statistically significant decrease of methylation frequency from start-point to end-point status (*p* < 0.05). Left panel: schematic illustration of *H. pylori* (-) L-MALT-related diseases in terms of CpG hypermethylation; right panel: schematic illustration of *H. pylori* (+) L-MALT related diseases in terms of CpG hypermethylation.

MALT lymphoma, a common low-grade B-cell lymphoma arising from a background of chronic inflammatory diseases at a number of mucosal sites, was first described by Isaacson and Wright in 1983 [30]. MALT lymphomas originating in the stomach are causatively linked to *Helicobacter pylori* infection, and eradication of the bacterium with antibiotics leads to long-term complete remission of the lymphoma in about 70% of patients [31]. Additional evidence links *Campylobacter jejuni* [32], *Chlamydia psittaci* [33], *Borrelia burgdorferi* [34], and hepatitis C virus [35,36] infection with MALT lymphoma of the small intestine, ocular adnexa, skin and splenic marginal zone, respectively. These organs are normally devoid of organized lymphoid tissue and lymphoma. MALT lymphomas arise from these sites as a result of chronic inflammatory or autoimmune disorders, such as *H. pylori*-associated chronic gastritis, lymphoepithelial sialoadenitis, Sjogren syndrome and Hashimoto thyroiditis [37,38]. The common karyotypic alterations that characterize MALT lymphoma include the trisomies of 3 and 18 [39,40] and the translocations t(11;18)(q21;q21) [41,42], t(1;14)(p22;q32) [43,44], t(14;18)(q32;q21) [39,45], and t(3;14)(p14.1;q32) [46,47], which commonly activate the NF-κB pathway. The most common translocation is t(11;18)(q21;q21), which results from the fusion of the *API2* (apoptosis inhibitor 2) and the *MALT1* (MALT lymphoma-associated translocation) genes [48,49]. Clonal identities of the immunoglobulin heavy chains between low-grade MALT lymphomas and coexisting diffuse large B-cell lymphoma (DLBCL) have been found in a considerable number of patients, indicating that low-grade MALT lymphoma progresses to high-grade malignancy composed of large-sized lymphoma (high grade MALT lymphoma) [50,51]. The detailed molecular mechanism of the progression to high-grade lymphoma has not been elucidated. Recently, we have demonstrated the average number of methylated genes was significantly greater in gastric lymphomas with *H. pylori* infection as compared to normal controls (*p* < 0.001). Concordant promoter hypermethylation of multiple genes, *i.e.*, the CpG island methylator phenotype (CIMP), was observed in 93.3% (14/15) of DLBCL, 100% (5/5) of high-grade MALT lymphomas, and 61.9% (13/21) of MALT lymphomas, in contrast, CIMP was not found in the healthy control group (0%) [52]. The average number of methylated genes and the CIMP incidence significantly increased with *H. pylori* infection. Furthermore, aberrant CpG methylation of specific genes, such as *P16*, *MGMT*, *DAPK*, *KIP2*, *H-cadherin (HCAD)* and *MINT31*, was consistently associated with *H. pylori* infection and lost after eradication therapy (Figure 1 and 2) [52]. These findings strongly suggest that *H. pylori* infection causes the aberrant DNA hypermethylation of specific genes and induces CIMP, which is an important epigenetic mechanism for the development and progression of gastric MALT lymphoma. Additionally, these findings provide new sensitive epigenetic markers to detect early stage MALT lymphoma and progression status of the diseases, knowledge of the epigenetic changes that occur in the genome of host and *H. pylori* should provide us with markers for following cancer progression, as well as new tools for cancer therapy. In several studies, aberrant DNA hypermethylation in gastric biopsies from *H. pylori*-positive patients has been shown to correlate with higher risk of gastric cancer [53,54]. Aberrant DNA hypermethylation is frequently associated with chronic inflammation, as observed in non-cancerous adjacent tissues of patients with inflammation-associated cancers, which should be further investigated in the context of *H. pylori* infection.

## 3. Epigenetic Alterations Induced by Virus Infection

A number of infectious agents, mainly viruses, have been reported to associate with human malignancies such as Epstein-Barr virus (EBV), human T lymphotropic virus type-I (HTLV-I), human T lymphotropic virus type-II (HTLV-II), hepatitis viruses (hepatitis B virus (HBV) and hepatitis C virus (HCV)), human papilloma virus (HPV), polyoma viruses (JC virus, BK virus, SV40) and Kaposi's sarcoma-associated herpesvirus/human herpesvirus-8 (KSHV/HHV-8). EBV has been associated with Burkitt's lymphoma and diffuse large B-cell lymphoma (DLBCL), NK/T lymphoma, nasopharyngeal carcinoma and Hodgkin's disease [55-57]. HTLV-I has been associated with adult T-cell leukemia/lymphoma (ATLL) [58-60], HTLV-II with hairy cell leukemia [61-63], HHV-8 with Kaposi's sarcoma and primary effusion lymphomas [64,65], HBV and HCV with hepatocellular carcinoma (HCC) [66,67], HPV with cervical carcinoma [68,69] and JCV with brain and colon cancer [70,71]. The molecular mechanisms by which these infectious agents contribute to the carcinogenesis and lymphomagenesis are not clear. However, some evidences discussed below suggest the important role of epigenetic changes and aberrant DNA methylation in the onset and progression of malignancies associated with infectious agents.

**Figure 3.**
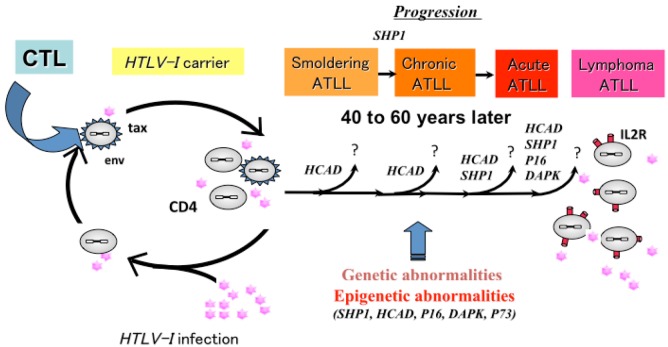
Natural course from the infection of human T lymphotropic virus type-I (HTLV-I) to onset and progression of adult T-cell leukemia/lymphoma (ATLL). Accumulation of genetic and epigenetic changes in host and virus genome during long latent period induce onset of ATLL.

ATLL is an aggressive malignant disease of CD4-positive T lymphocytes caused by infection with HTLV-I [58-60]. HTLV-I causes ATLL in 3-5% of infected individuals after a long latent period of 40–60 years [72]. Advanced acute ATLL has poor prognosis. ATLL is divided into four stages: namely, smoldering, chronic, lymphoma and acute types [73]. The smoldering and chronic types are indolent, but the acute and lymphoma types are aggressive ATLL is characterized by resistance to chemotherapy and a poor prognosis [73,74]. Such a long latent period suggests that a multi-step leukemogenic/lymphomagenic mechanism is involved in the development of ATLL, although the critical events in the progression have not been well characterized. The pathogenesis of HTLV-I has been intensively investigated in terms of the viral regulatory protein HTLV-I Tax or Rex, which is supposed to play key roles in the HTLV-I leukemogenesis/lymphomagenesis as well as the recently discovered HTLV-I basic leucine zipper factor (HBZ) [74-76]. We and others have reported the progression mechanism of ATLL from various genetic aspects, including specific chromosome abnormalities [77-82], changes of characteristic HTLV-I Tax and Rex protein expression patterns [71] and aberrant expression of the *SHP1* [78,83], *P53* [84,85],*MELIS* [85], *DRS* [86] and *ASY/Nogo* [87] genes, although the detailed mechanism triggering the onset and progression of ATLL remains to be elucidated. Frequent epigenetic aberration of DNA hypermethylation associated with the *SHP1* gene silencing has been identified in a wide range of hematopoietic malignancies [83,88]. SHP1 is a nonreceptor type protein-tyrosine phosphatase, which acts as a negative regulator in hematopoietic cells. A decrease or loss of the *SHP1* gene expression may be related to the malignant transformation in lymphoma and leukemia cells [72,84]. Recently, we have reported that the number of CpG island methylated genes, including the *SHP1*, *P15*, *P16*, *P73*, *HCAD*, *DAPK* and *MGMT* genes, increased with disease progression and that aberrant hypermethylation in specific genes was detected even in HTLV-I carriers and correlated with progression to ATLL (Figure 3)[89]. CIMP was observed most frequently in lymphoma type ATLL and was also closely associated with the progression and crisis of ATLL. The high number of methylated genes and increase of CIMP incidence were shown to be unfavorable prognostic factors and correlated with a shorter overall survival with the Kaplan-Meyer analysis. These findings strongly suggest that the multi-step accumulation of aberrant CpG methylation in specific target genes and the presence of CIMP are deeply involved in the crisis, progression and prognosis of ATLL as well as the value of CpG methylation and CIMP for new diagnostic and prognostic biomarkers.

Integration-defective HIV-I was shown to increase DNMT1 expression, followed by the increased methylation of CpG islands in the promoter region of the *p16^INK4A^* and *IFN-gamma* genes to induce gene silencing [90,91]. The latent membrane protein 1 (LMP-1), one of the virus proteins of EBV, has been shown to be an oncoprotein with transforming activity [92]. LMP-1 activates DNMTs to initiate epigenetic alterations, followed by hypermethylation and gene silencing of the *E-cadherin* gene. Human epithelial cells expressing LMP-1 have been shown to have higher invasive activity in accordance with reduced expression of the *E-cadherin* gene [92,93]. It is quite interesting to make clear whether there is a direct link of HTLV-I induction of DNMTs to cause CIMP and hypermethylation of specific target genes, and how or what kind of virus molecules induce deregulation of epigenetical machinery. It may open new insight for the total understanding of the molecular mechanism of virus-induced lymphomagenesis and leukemogenesis.

## 4. Concluding Remarks and Perspective

Aberrant DNA methylation has been shown to be the most consistent molecular change in various kinds of neoplasia as well as virus or microbial infection-induced lymphoma/leukemia [94,95]. Epigenetic alterations, responding to the fine environmental changes and infection, might be useful as a marker prior to the onset and progression of lymphoma and leukemia. Thus, epigenetic markers may be crucial for identifying the risk of tumor development. Epigenetic changes, unlike genetic changes, can be easily reverted by the use of therapeutic interventions such as selective inhibitors against DNMTs and HDACs. DNMT inhibitors such as 5-aza-2′-deoxycytidine or 5-azacytidine have been approved in myelodysplastic syndrome (MDS) and acute myelogenous leukemia (AML), whereas the histone deacetylase inhibitors (HDIs) including vorinostat, romidepsin, panobinostat, belinostat, and entinostat have been shown to be active in cutaneous and peripheral T-cell lymphoma [96]. Using virus, and microbial genomes, such as HTLV-I and *H. Pylori*, and host cellular methylated genes as therapeutic markers, another kind of intervention may be developed to prevent onset and progression of lymphomas and leukemias from carrier stage. We are now in a new era of epigenetics. Technologies for the global analyses of the epigenome are developing with remarkable speed such as ChIP-on-chip (chromatin immunoprecipitation method with micro-array) and ChIP-sequence with deep sequencing by next generation sequencers for mapping global methylation and chromatin modifications, which may provide the new landscape of infection-induced alterations, information about the dynamic nature of microbe-host interactions and the human epigenome itself with relationship to the various kind of diseases.
